# Current Knowledge and Challenges in the Clinical Management of Spontaneous Coronary Artery Dissection (SCAD): A Case Series

**DOI:** 10.7759/cureus.61847

**Published:** 2024-06-06

**Authors:** Jack Jnani, Ilja Dejanovic, Christian Leung, Avneet Singh

**Affiliations:** 1 Internal Medicine, North Shore University Hospital, Manhasset, USA; 2 Cardiology, North Shore University Hospital, Manhasset, USA

**Keywords:** congestive heart failure, percutaneous coronary intervention, conservative management, iatrogenic coronary artery dissection, spontaneous coronary artery dissection (scad)

## Abstract

Spontaneous coronary artery dissection (SCAD) is a rare condition in which there is coronary dissection that is not due to atherosclerosis or iatrogenic causes. It is more common in young women and is associated with risk factors such as the peripartum period and connective tissue disorders.

We present five unique cases of SCAD to illustrate the variety of presentations and clinical management. The youngest and oldest patients in our series were 34 and 63 years old, respectively. The majority of our patients (60%) were of African American ethnicity. Two of the patients in the case series developed a new-onset congestive heart failure, and one patient had an iatrogenic complication after intervention. The majority of the patients were treated with conservative medical management (60%), while the others were treated with primary percutaneous coronary intervention (PCI).

SCAD is a rare but life-threatening disease that may have varying presentations and precipitating risk factors. As demonstrated in our case series, SCAD may present atypically, and clinicians should maintain a high degree of suspicion in a relevant presentation. Treatment of SCAD may involve conservative management, primary PCI, or coronary artery bypass grafting (CABG) depending on the case. Clinicians may also have to address complications from SCAD, such as cardiomyopathy, that may arise.

## Introduction

Spontaneous coronary artery dissection (SCAD) is a rare condition characterized by coronary artery dissection that is not due to atherosclerosis or iatrogenic causes [1]. It is more common in young women and is associated with risk factors including the peripartum period and connective tissue disorders, such as Ehlers-Danlos syndrome (EDS) [1]. Management of SCAD may prove challenging, especially with underlying arteriopathy or connective tissue disorders, and patients may experience a host of sequelae, such as a reduction in cardiac function. In this case series, we illustrate differing presentations of SCAD, as well as some of the challenges and complications of its management.

## Case presentation

Case 1

**Figure 1:**
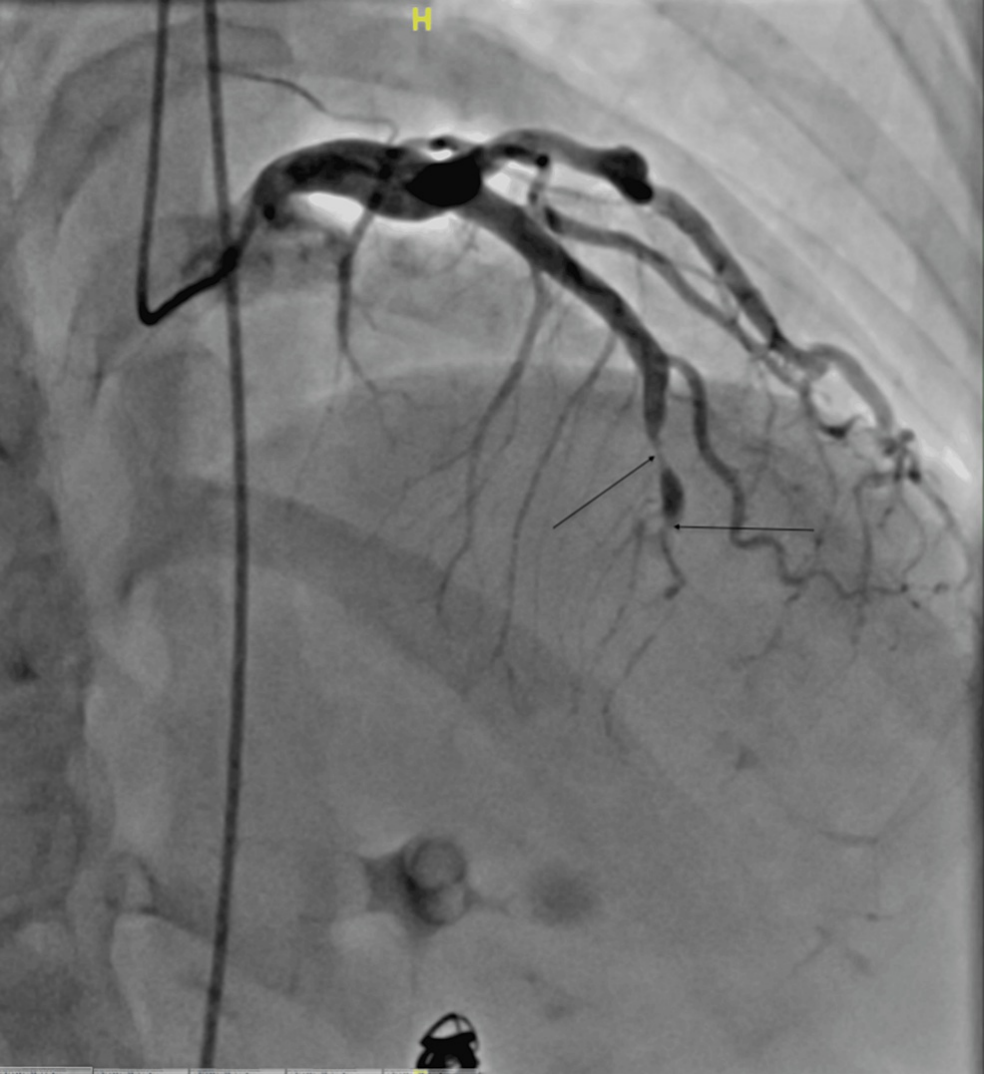
Case 1: cardiac catheterization showing 90% mid-LAD occlusion and 100% distal LAD occlusion likely from SCAD LAD: left anterior descending artery; SCAD: spontaneous coronary artery dissection

**Figure 2:**
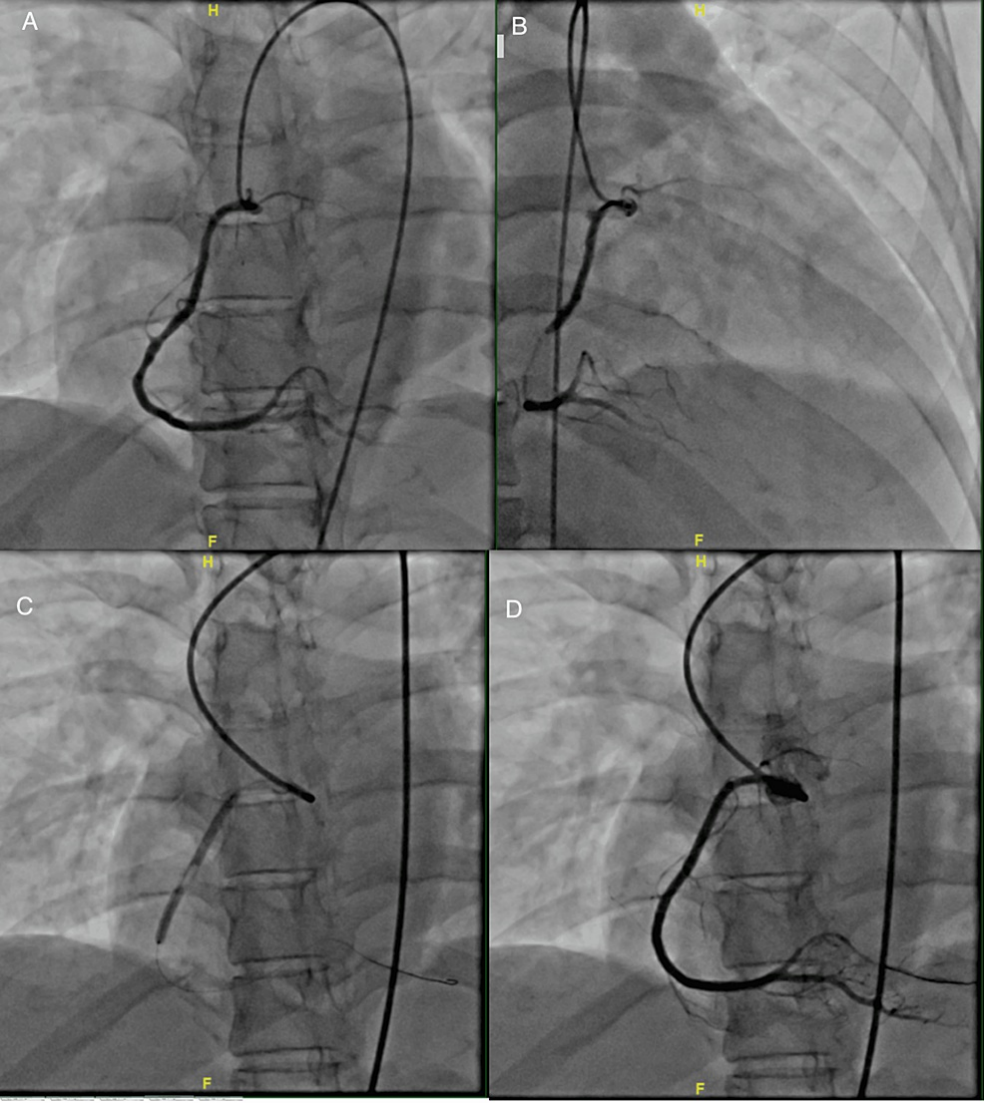
Case 1 (A-D): series of catheterization images showing the occlusion of the proximal RCA due to SCAD. This was complicated by an iatrogenic spiral dissection during guidewire manipulation resulting in a 100% occlusion which was subsequently rescued with three DES with the restoration of coronary flow RCA: right coronary artery; SCAD: spontaneous coronary artery dissection; DES: drug-eluting stent

A female in her 30s with vascular EDS due to a pathogenic COL3A1 genetic variant (which encodes the pro-alpha 1 chain of type III procollagen) complicated by splenic, iliac, superior mesenteric, and anterior communicating arterial aneurysms along with chronic left internal carotid artery dissection presented after a caesarian section six days prior due to abdominal pain for the past three days. She endorsed epigastric abdominal pain without radiation, nausea, vomiting, and diarrhea. On admission, her vitals were stable. Electrocardiogram (ECG) showed T-wave inversions in leads V2-V4 consistent with Wellens syndrome. Transthoracic echocardiography (TTE) showed an ejection fraction (EF) of 48% (54% on a prior TTE), moderate mitral and tricuspid regurgitation, mild segmental left ventricular systolic dysfunction, stage III diastolic dysfunction, and complete akinesis of the apical and inferior walls of the heart. Laboratory workup was significant for leukocytosis to 14.08 K/uL and elevated cardiac enzymes including troponin T of 478 ng/L, troponin I of 2120.6 ng/L, creatine kinase of 417 U/L, and creatinine kinase-MB of 17.4 ng/mL. Angiography showed 100% stenosis of the distal left anterior descending artery (LAD), 90% stenosis of the mid-LAD (Figure 1), 30% stenosis of the proximal left circumflex artery, and 40% stenosis of the first obtuse marginal artery (OM1). The 90% mid-LAD occlusion and 100% distal LAD occlusion were presumed to be secondary to SCAD. Two drug-eluting stents (DES) were placed in the distal LAD. Angiography also showed an occlusion of the proximal right coronary artery (RCA), but during guidewire manipulation, an iatrogenic spiral dissection occurred resulting in a 100% occlusion which was subsequently rescued with three DES (Figure 2A-2D). Following revascularization, she was further managed medically with dual antiplatelet medications, a beta-blockade medication, and angiotensin receptor blocker therapy. After revascularization, the patient was noted to have a large right groin hematoma extending from the lateral thigh to the pubic region, but hemostasis was achieved. She made a successful recovery and was discharged to home with appropriate cardiology and vascular follow-up appointments.

Case 2

**Figure 3:**
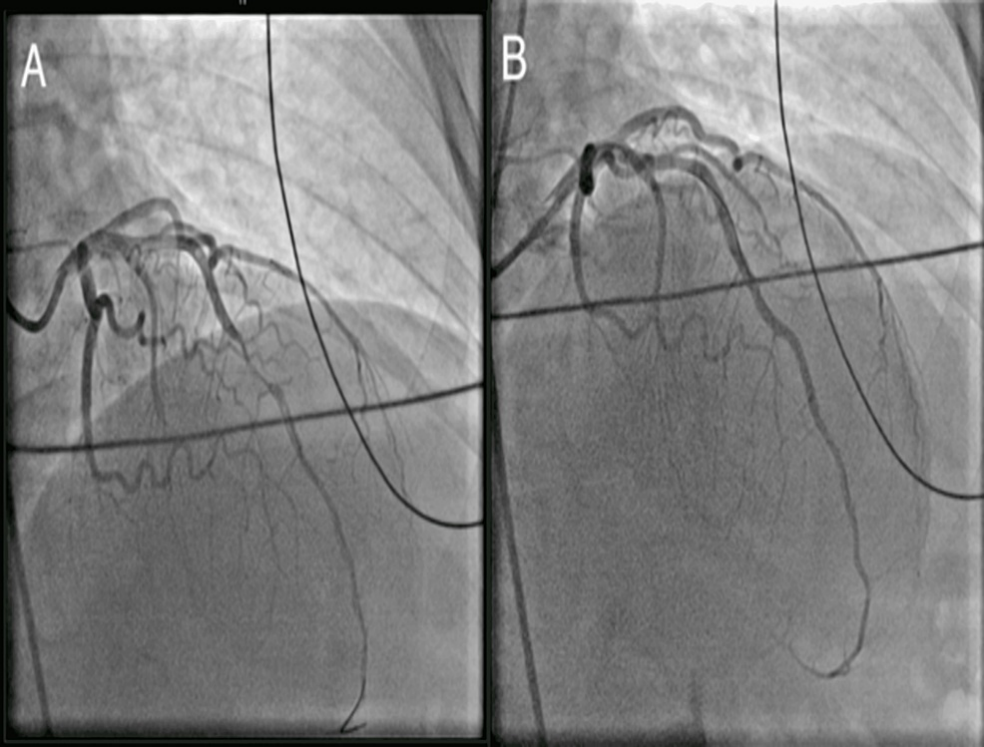
Case 2: catheterization showing a 100% stenosis of the mid-LAD from SCAD before (A) and after (B) intervention LAD: left anterior descending artery; SCAD: spontaneous coronary artery dissection

A woman in her 40s with a history of hypothyroidism, anxiety, cholecystectomy two months ago, and a family history of coronary artery disease in both her parents presented with severe acute chest pain. She had an episode of exertional chest discomfort after hiking four days ago that spontaneously resolved. However, she later had an episode of non-exertional chest pain that began while she was sitting down and has been persistent. Her pain was substernal, radiating to the back, rated as 10/10, and described as a sharp or jabbing pain that is worse with sitting up. She also described right upper extremity tingling and numbness, palpitations, headaches, and shortness of breath. On admission, she was tachycardic to 110 beats/minute and hypertensive to 154/99 mmHg, with an oxygen saturation of 99% on room air. Labs were significant for a troponin T of 1115 ng/L, aspartate aminotransferase of 205 U/L, alanine aminotransferase of 104 U/L, total cholesterol of 207 mg/dL, low-density lipoprotein (LDL) cholesterol of 130 mg/dL, creatine kinase-MB of 97 ng/mL, creatine kinase of 887 U/L, and C-reactive protein of 21.9 mg/L. CT angiography showed no acute aortic dissection or pulmonary embolism. ECG on admission showed evidence of anterior wall ischemia. Echocardiography on admission showed an EF of 30% and severe segmental left ventricular systolic dysfunction involving the mid-to-distal septal, apical, mid-to-distal lateral, and mid-to-distal anterior wall hypokinesis, with mostly preserved function in the basal segments of the heart. Right ventricular function was normal. Catheterization revealed a 100% stenosis of the mid-LAD (Figure 3A) suspected to be from a type 2a coronary artery dissection. Balloon angioplasty was performed, and a DES was placed in the mid-LAD with improvement in flow (Figure 3B). She was discharged on dual antiplatelet therapy for recent stenting, atorvastatin, and goal-directed medical therapy with metoprolol succinate and losartan for the resulting cardiomyopathy and heart failure with reduced EF (HFrEF). Echocardiography two months later showed significant improvement in left ventricular function following treatment, with an EF of 50% and mild segmental left ventricular systolic dysfunction. 

Case 3

**Figure 4:**
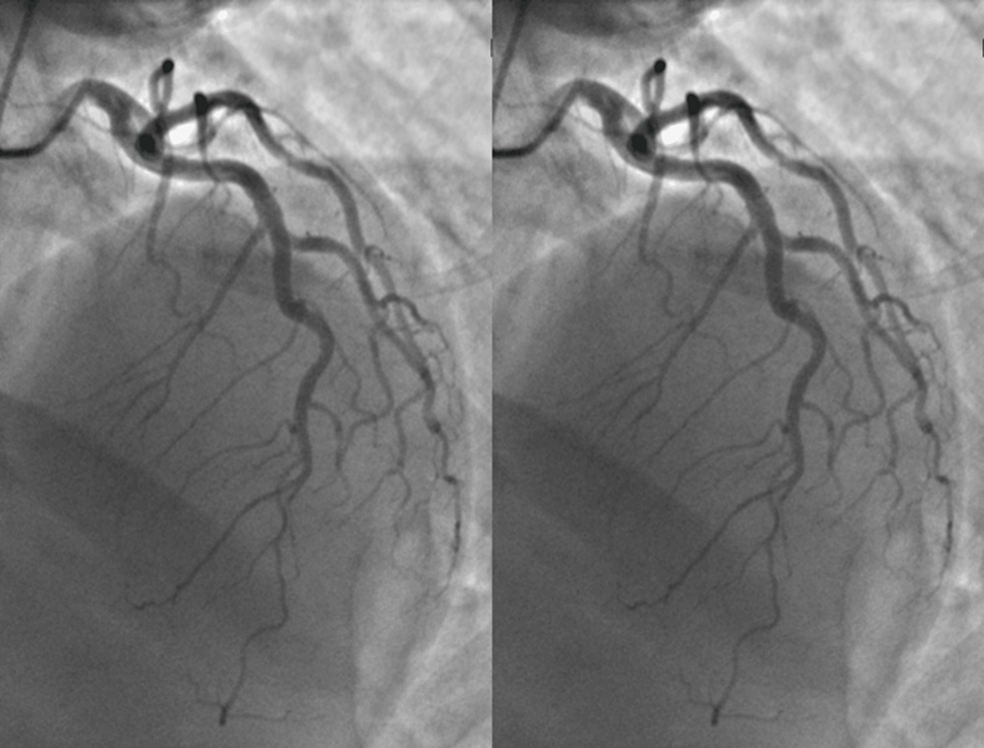
Case 3: series of catheterization images showing distal LAD SCAD LAD: left anterior descending artery; SCAD: spontaneous coronary artery dissection

A man in his 50s with a history significant for schizophrenia, hypertension, hyperlipidemia, type 2 diabetes, and active cocaine and tobacco use presented for chest pain. He last used cocaine one day prior to admission. The chest pain was sudden in onset, left-sided, non-radiating, and constant, without relief from rest. He was hemodynamically stable on admission, and physical examination was unrevealing. Laboratory data on admission was notable for elevated troponin T and positive urine toxicology for cocaine. ECG demonstrated normal sinus rhythm, with ST-segment elevations in the anterolateral leads. Chest X-ray was without abnormality. While being evaluated in the emergency department (ED), he had persistent chest pain and rising troponin levels, so he was taken for cardiac catheterization, which demonstrated a small-caliber distal LAD with diffuse disease (Figure 4) that was unresponsive to nitroglycerin administration. The remainder of the coronary vasculature showed mild atherosclerotic disease. The clinical picture was consistent with SCAD. The decision was made to manage his disease medically. Subsequent TTE demonstrated normal EF without regional wall motion abnormalities. The patient's chest pain improved with medical management, and he was discharged without complications.

Case 4

**Figure 5:**
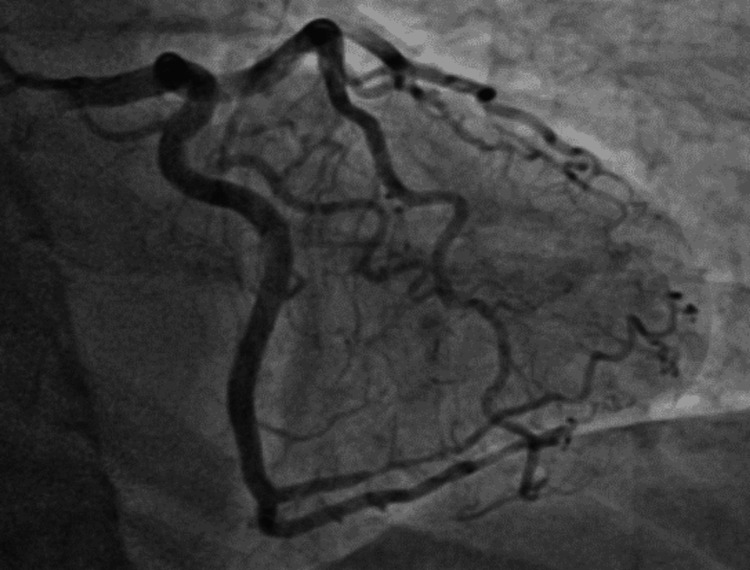
Case 4: catheterization image showing OM2 SCAD OM2: obtuse marginal 2; SCAD: spontaneous coronary artery dissection

A woman in her 50s with a medical history of hyperlipidemia, asthma, and myocarditis presented to the ED with pressure-like chest and epigastric pain radiating to the left jaw for three hours, not exacerbated by exertion. She denied associated nausea or diaphoresis. She had presented to the ED a few days ago for similar symptoms. Workup at that time showed elevated cardiac enzymes but a non-ischemic ECG. Since the patient's chest pain resolved, she was discharged to home. However, chest pain recurred, so she returned for further medical attention. On this presentation, vital signs were also stable, and physical exam was unremarkable. Troponin T was mildly elevated, but downtrending, and ECG demonstrated sinus tachycardia without ST-segment deviations. Given the recurrent nature of the patient's chest pain, the decision was made to perform cardiac catheterization, which demonstrated moderate diffuse stenosis of the obtuse marginal 2 (OM2) coronary segment, suggestive of SCAD (Figure 5). The lesion had normal flow and the vessel was small, so no intervention was performed. The patient was then managed medically, including with anticoagulation with a continuous heparin infusion. Echocardiography at this time demonstrated a normal EF without regional wall motion abnormalities. The patient had improvement in her chest pain and was discharged to home without complications. 

Case 5

**Figure 6:**
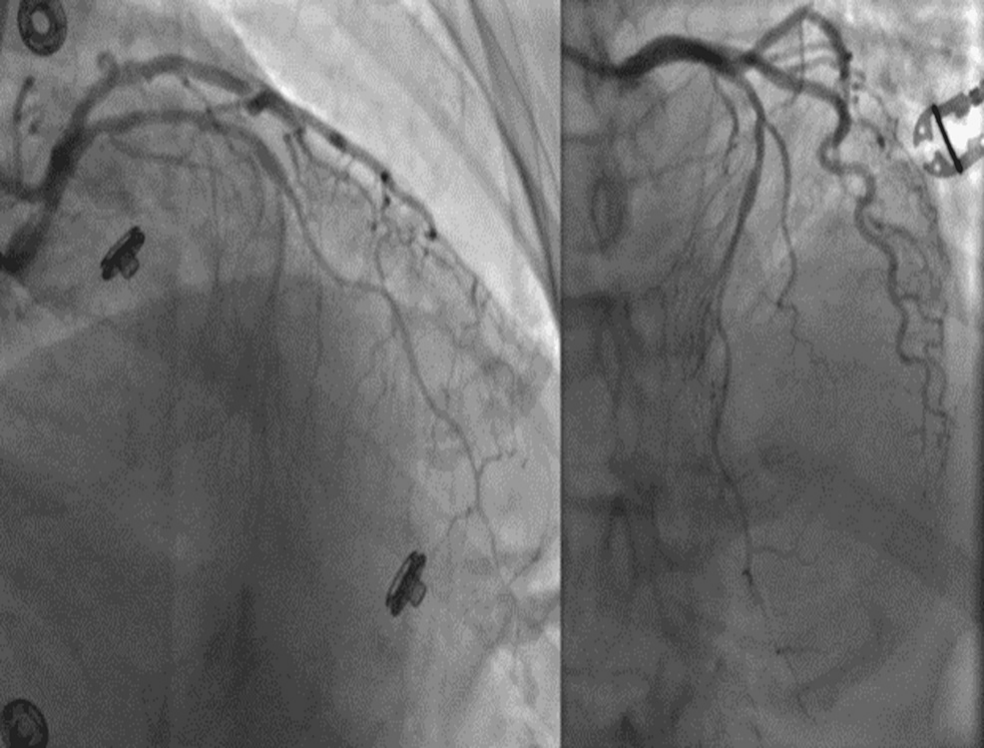
Case 5: series of catheterization images showing proximal and mid-LAD SCAD LAD: left anterior descending artery; SCAD: spontaneous coronary artery dissection

A woman in her 60s with a history of hepatitis B presented with severe crushing chest and epigastric pain radiating to the left shoulder, with associated hand numbness, nausea, and vomiting. The pain was worse with exertion and not relieved by rest. On presentation to the ED, she was noted to be hypertensive, though all other vital signs were stable. Physical exam was unremarkable. ECG demonstrated normal sinus rhythm with ST elevations in leads V3-V6. Laboratory data was remarkable for elevated cardiac enzymes; repeat blood work demonstrated an increase in cardiac enzymes. Chest X-ray revealed no abnormalities. Given the evidence of ongoing myocardial ischemia, the patient was taken for cardiac catheterization, which demonstrated diffuse disease of the proximal and mid-LAD (Figure 6), with optical coherence tomography revealing SCAD. As there was preserved flow across the lesion, the decision was made to manage the patient medically. She was treated with a continuous heparin infusion, as well as aspirin, clopidogrel, lisinopril, and metoprolol succinate. Subsequent TTE demonstrated an EF of 40% and a hypokinetic left ventricular apex. The patient was discharged in a stable condition.

A summary and comparison of all five SCAD case presentations is included in Table 1. 

**Table 1:** A comparison of the five SCAD case presentations, clinical management, and complications BMI: body mass index; ECG: electrocardiogram; TTE: transthoracic echocardiography; EF: ejection fraction; LV: left ventricular; LAD: left anterior descending artery; RCA: right coronary artery; SCAD: spontaneous coronary artery dissection; DES: drug-eluting stent; OM2: obtuse marginal 2

	Case 1	Case 2	Case 3	Case 4	Case 5
Age	34 years	45 years	56 years	52 years	63 years
Sex	Female	Female	Male	Female	Female
Race	African American	Caucasian	African American	African American	Asian
BMI (kg/m^2^)	17.2	33.2	29.8	Not recorded	30.2
Significant medical history	Vascular Ehlers-Danlos syndrome	Hypothyroidism	Schizophrenia	Myocarditis	Hepatitis B
Significant social history	No alcohol, smoking, recreational substances	No alcohol, smoking, recreational substances	Cocaine use	No alcohol, smoking, recreational substances	No alcohol, smoking, recreational substances
Presenting symptom	Epigastric abdominal pain, nausea, and vomiting after childbirth	Chest pain after a hike	Chest pain one day after cocaine use	Recurrent chest pain requiring multiple hospital admissions	Exertional chest pain
Laboratory findings	Troponin T 478 ng/L, troponin I 2120.6 ng/L, CKMB 17.4 ng/mL	Troponin T 1115 ng/L, CKMB 97 ng/mL	Troponin T 154 ng/L	Troponin T 66 ng/L	Troponin T 412 ng/L
ECG	Wellens syndrome	Anterior wall ischemia	ST elevation in anterolateral leads	Sinus tachycardia, no ischemic changes	ST elevations in leads V3-V6
TTE	EF 48%, LV wall dysfunction	EF 30%, multiple regional wall abnormalities	Normal	Normal	EF 40%, hypokinetic LV apex
Cath findings	LAD and RCA SCAD	Mid-LAD SCAD	Distal LAD SCAD	OM2 SCAD	Proximal and mid-LAD SCAD
Management	DES to LAD and RCA	DES to LAD	Medical	Medical	Medical
Complication	Iatrogenic spiral dissection of RCA	New-onset heart failure	None	None	New-onset heart failure

## Discussion

SCAD is the disruption and separation of the coronary artery wall that is not iatrogenic or due to atherosclerosis. SCAD is a rare but important cause of myocardial infarction and can lead to coronary artery obstruction through intramural hematoma formation or the disruption of the intima at the site of the true lumen [1]. While SCAD classically affects young women and presents with anginal chest pain, the presentation can be variable as we have highlighted in this case series. While classic risk factors and symptoms may facilitate the diagnosis of SCAD, lack of clinician familiarity with the condition and lack of classic symptoms or risk factors in the patient's presentation may lead to an underdiagnosis of SCAD.

SCAD occurs predominantly in women and is frequently seen in the postpartum period as case 1 demonstrated. In a retrospective review of 63 patients with non-atherosclerosis-related SCAD, 94% were women, 35% presented with symptoms of acute coronary syndrome (ACS) in women less than 50 years old, and 8.1% were pregnancy-related SCAD [2]. Furthermore, in a retrospective study of 150 cases of pregnancy-associated myocardial infarction with a mean age of 34 years old, SCAD was present in up to 43% of cases [3].

SCAD has been associated with a variety of underlying arteriopathy, including fibromuscular dysplasia (most common), Marfan syndrome, and vascular EDS, as seen in our first case [1].

Vascular EDS is a rare genetic condition in which there is a mutation affecting collagen matrix formation resulting in weakened blood vessels. This can predispose patients to ruptures and dissections of major blood vessels. SCAD may be precipitated by a significant stress, such as a caesarian section.

SCAD is a rare complication of EDS, though this may be due to a low prevalence of reported cases. In a recent study of 26 cases of vascular EDS-related SCAD, the authors reported 30% mortality either with surgical repair or without intervention [4].

SCAD has a variety of risk factors including fibromuscular dysplasia, pregnancy, inherited arteriopathy (case 1), systemic inflammatory diseases, and precipitating factors including intense exercise (case 2), recreational drug use (case 3), intense emotional stress, and exogenous hormone use [1]. 

Unlike atherosclerosis-related ACS, SCAD is managed differently. In clinically stable patients with no signs of hemodynamic instability, conservative management is preferred, as spontaneous healing is possible. In a retrospective study of 131 patients with SCAD who had repeat coronary angiography, spontaneous healing occurred in 88.5% of cases within 35 days and 100% of cases after 35 days [5]. In a review of 24 observational studies with 1720 patients with SCAD, a conservative approach was associated with similar clinical outcomes including all-cause death, cardiovascular death, SCAD recurrence, and lower target vessel revascularization rates compared to an invasive strategy [6]. Therefore, the American College of Cardiology (ACC) guidelines currently recommend conservative therapy in patients who are stable with no high-risk features such as SCAD involving the left main coronary, ostial LAD, >2 proximal coronaries, ventricular tachycardia/fibrillation, ongoing ischemia, and cardiogenic shock [1]. Similarly, the American Heart Association (AHA) currently recommends conservative management in patients who are clinically stable with no high-risk anatomy. In clinically stable patients with left main or severe proximal two-vessel dissection, the AHA guidelines state that coronary artery bypass grafting (CABG) should be considered though conservative management may be reasonable. However, in cases of active ischemia or hemodynamic instability, the AHA recommends percutaneous coronary intervention (PCI) if feasible or CABG based on local expertise [7]. 

However, conservative therapy may not be appropriate in certain patients, such as in patients with vascular EDS-related SCAD, in whom normal healing by collagen matrix formation is impaired. Additionally, in clinically stable patients with left main plus LAD or left circumflex dissection, ostial LAD dissection, or severe proximal two-vessel dissection, CABG may be reasonable. In patients with isolated left main dissection, PCI may be reasonable. Furthermore, in patients with hemodynamic instability, or active ischemia, PCI or urgent CABG may be necessary [1].

Intervention has its risks, especially among patients with SCAD. First, the coronary arteries may be inherently weak because of underlying arteriopathy (such as EDS) putting them at higher risk of iatrogenic dissection and extension of dissection with intervention (as case 1 demonstrated). Second, balloon dilatation and stenting may lead to the extension of coronary dissection or propagate the intramural hematoma along the affected coronary vessel leading to worsening obstruction. Next, the process of stenting may be technically challenging due to the presence of true and false lumens. Lastly, SCAD most commonly affects the distal coronary segments which may be too small or too distal for stenting.

In a Mayo Clinic series of 189 patients with SCAD, technical failure with PCI occurred in 53% of patients, emergency CABG was required in 13% of cases, and there was one death [8]. In a Vancouver study of 168 patients with SCAD, technical failure occurred in 36%, stent thrombosis in 6%, and need for emergency CABG in 12% of cases [9]. Furthermore, connective tissue disorders, such as vascular EDS, may have an even higher risk of complication.

Medical management of SCAD involves managing hypertension and other complications that may arise, such as cardiomyopathy. In a large prospective study of 327 SCAD patients at Vancouver General Hospital, hypertension increased the risk of recurrent SCAD, whereas beta-blockers seemed to be protective [10], suggesting a possible role for beta-blockers in SCAD management.

An important complication of SCAD is cardiomyopathy. In a large prospective cohort study of 277 patients with SCAD, the majority of patients presented with wall motion abnormalities but had a relatively normal EF (unlike case 2 and case 5). Over half of the patients had subsequent normalization of wall motion abnormalities and EF on follow-up assessment [11].

In summary, SCAD can have a variety of different presentations, risk factors, and complications. In the first case, we discussed a woman with inherited arteriopathy (reported prevalence in cohort studies 1.2-3%) [1] who developed SCAD after a recent delivery of her child (the precipitating risk factor). However, she had a relatively atypical presentation (abdominal pain, nausea, and vomiting) and a significant iatrogenic complication highlighting the complexity that SCAD could entail. 

In the second case, we discussed a woman who developed SCAD after a precipitating factor of intense physical activity after her hike. She went on to develop congestive heart failure which improved after cardiac intervention. However, some patients with SCAD may not have the same favorable outcome.

In the third case, we presented a male with schizophrenia who developed SCAD following a significant precipitating factor which was his recreational drug use of cocaine. Although cocaine itself could lead to acute myocardial infarction, tachyarrhythmias, and myocarditis, SCAD should also be on the differential as this case demonstrates.

In the fourth case, we discussed a woman who had a history of myocarditis and had an admission for SCAD after recurring admissions for chest pain. Systemic inflammatory diseases are prevalent in 1-8.9% of SCAD cases [1]. Therefore, this case demonstrates the importance of keeping inflammatory conditions in mind as risk factors for SCAD development.

In the final case, we discussed a patient with a history of hepatitis B virus who developed SCAD. Hepatitis B is not typically associated with SCAD. Therefore, this case may demonstrate a case of SCAD where it was spontaneous with no obvious acute triggers. She also went on to develop subsequent congestive heart failure, which can be seen with SCAD and was treated accordingly.

## Conclusions

SCAD is a rare but important cause of acute coronary syndrome in young and middle-aged women without traditional cardiovascular risk factors. It is often underdiagnosed but represents an important cause of pregnancy-associated myocardial infarction. The etiology of SCAD is likely multifactorial but has been frequently reported following a significant physical or emotional stressor. As shown in our case series, SCAD may have differing clinical presentations and precipitating risk factors. Treatment of SCAD may vary depending on the specifics of the case and can involve conservative management, primary PCI, or CABG. There may also be complications from SCAD such as congestive heart failure, ventricular tachyarrhythmias, or iatrogenic complications that need to be addressed. A multidisciplinary approach is crucial in the care and management of patients with SCAD.
